# Comparison of four matrixes for diluting insulin in routine clinical measurements

**DOI:** 10.1002/jcla.23396

**Published:** 2020-06-07

**Authors:** Honglei Li, Danchen Wang, Xiuzhi Guo, Liangyu Xia, Qiong Wu, Xinqi Cheng

**Affiliations:** ^1^ Department of Laboratory Medicine Peking Union Medical College Hospital Chinese Academy of Medical Sciences Beijing China; ^2^ Department of Laboratory Medicine Affiliated Hospital of Chifeng University Inner Mongolia China

**Keywords:** diluent, insulin, matrix, recovery

## Abstract

**Objective:**

In our laboratory, 2.36% (6626/280765) samples obtained for insulin evaluation have serum insulin concentrations higher than 300 mU/L, resulting in curves outside the linear range in the insulin release test (IRT). Accordingly, using appropriate dilution protocols to determine insulin concentration accurately is important. Here, we compared the effectiveness and economy of four different solutions for diluting high‐insulin serum in routine clinical measurements.

**Method:**

Residual serum samples with high‐insulin concentrations ranging from 200 to 300 mU/L were collected in Peking Union Medical College Hospital from August to November 2017. Four different matrixes including a Siemens original diluent, pure water, 0.9% NaCl, and low‐insulin serum (labeled as A to D, respectively) were used to dilute the serum in the ratios of 1:2, 1:5, and 1:10.

**Results:**

We found that the linear correlation coefficients of A to D were higher than 0.9. The recovery rates of A to D were 86.4%–104.0%, 73.2%–99.3%, 76.4%–101.3%, and 84.2%–99.7%, respectively. We conclude that the use of 0.9% NaCl, pure water, or low‐insulin serum to dilute high‐serum insulin (>300 mU/L) is feasible and cost‐effective.

**Conclusion:**

We recommend a dilution factor of 1:5 on a Siemens ADVIA Centaur XP^®^ instrument. The clinically reported range was 0.5‐1500 mU/L. For specific samples (>1500 mU/L), we recommended using low‐insulin serum samples for dilution.

**Abbreviations**
CLSI: Clinical Laboratory and Standard Institution
CV: coefficient of variation
IDF: International Diabetes Federation
IRT: insulin release test
OGTT: oral glucose tolerance test

## 1 INTRODUCTION

Insulin is a peptide hormone synthesized and secreted by islet β cells. The main function of insulin is to regulate the concentration of glucose in the blood, and secondly, it plays an important role in the metabolism of lipids and proteins. From a clinical perspective, the measurement of serum insulin concentration provides useful information for the diagnosis of insulin deficiency and insulin resistance,^1^, ^2^ particularly in diabetes, neonatal hyperinsulinemia hypoglycemia, insulinoma, and polycystic ovary syndrome.^3^, ^4^, ^5^ Moreover, in addition to its therapeutic application in diabetes, insulin therapy can improve lipid metabolism and decrease mortality for myocardial infarction patients.^7^, ^8^, ^9^

Physiological insulin therapy with insulin analogs is now relatively simple to use and is associated with fewer episodes of hypoglycemia in diabetics.^6^ To optimize its use, it is important to predict the degree of postprandial hyperglycemia and the likely response to prandial insulin.^2^, ^7^, ^9^ Accurate measurement of insulin is also helpful for evaluating insulin therapy compliance and suspected overdose,^10^ which is particularly important in diabetic mothers.^11^

A useful test of endogenous insulin function is the IRT or oral glucose tolerance test (OGTT). It involves the administration of oral glucose to a fasting patient to increase blood glucose and stimulate the β cells to release insulin. Serum insulin concentrations are measured at fasting, 0.5, 1, 2, and 3 hours after taking sugar. Normal human insulin secretion often peaks at 60 minutes after taking sugar, and then returns to normal concentration within 2 hours.^6^ However, when the patient has severe insulin resistance due to polycystic ovary syndrome, obesity, type 2 diabetes, or other illness, the serum insulin concentration at each of the above points may exceed the upper limit of the detection system. If a specific value cannot be detected at this time, the patient's insulin peak time cannot be determined, and multiple peaks are compared with the increase in fasting insulin levels. Otherwise, we also obtained the clinical laboratory real data to manifest the importance of an insulin dilution study.

Since 2012, our laboratory has worked on developing the serum insulin test. Of 280765 test reports from the previous 7 years, we found that 2.36% (6626/280765) showed OGTT curves produced results that exceeded the limit of detection. This rate was quite consistent through these years (2.66% [611/23011] in 2012, 3.01% [838/27815] in 2013, 2.98% [1023/34285] in 2014, 2.62%, [1101/41979] in 2015, 3.23% [1220/37805] in 2016, 1.96% [802/41009] in 2017, 1.29% [524/40574] in 2018, and 1.45% [497/34287] in 2019). It is estimated that 69 samples per month (2.3 samples per day) exceed the upper limit of linearity. At present, the Siemens detection system used in our laboratory provides an original diluent for insulin dilution measurement and can realize automatic dilution, eliminating the error introduced by manual dilution. However, the minimum dilution package of the original diluent is 2 × 10 mL. We calculated that using the original diluent (validity: 21 days 10 mL; dilution factor: 1:5; 40 μL) to dilute high‐insulin serum could result in the wastage of 22.72% of the original diluent.

To obtain accurate serum insulin concentration at all time points of the OGTT, and thereby extend the applicability of this test to more patients, it is necessary to issue a dilution measurement report on recalcitrant specimens. The purpose of this study was to investigate the feasibility of using other diluents to replace the original diluent provided by the manufacturer to dilute samples with insulin concentrations above the upper limit of linearity. Considering the impact of health economics, we aimed to find a low‐cost and effective diluent for routine clinical working. Thus, we evaluated the effects of four different diluents including the original Siemens diluent, pure water, 0.9% NaCl, and low‐insulin serum to dilute high‐insulin samples. This study aimed to provide an enhanced protocol for routine clinical work to ensure accurate results with an efficient test.

## 2 MATERIAL AND METHODS

### 2.1 Precision

To evaluate precision of insulin measurements, we used quality controls (Lyphochek^®^ Immunoassay Plus Control) including three different concentrations for verification. Pools with three different concentrations were separately dispensed into 25 portions and then frozen at −80°C. Before testing, each sample was equilibrated to room temperature and mixed. According to Clinical Laboratory and Standard Institution (CLSI) EP15‐A, the precision of our method was validated. The repeatability and within‐laboratory precision (coefficient of variation, CVs) were calculated for four replicates of three concentrations over 5 days.

### 2.2 Diluent preparation

In this study, four dilutions were used, including original Siemens diluent (main components: potassium thiocyanate buffer and sodium sulfide), pure water (Millipore), 0.9% NaCl, and low‐insulin serum pools (serum samples without bilirubin, hemolysis, and lipemia) (labeled as A to D, respectively). The concentration of low‐insulin serum samples ranging from 0.5 to 2 mU/L was compared.

Low‐insulin serum samples (n = 16) were obtained from clinical residual serum between August 22 and November 29, 2017 and stored at −80°C until use. Before analysis, all of the low‐insulin serum samples were brought up to room temperature and mixed together. The total amount of low‐insulin serum pool obtained was 8 mL. The average concentration of the low‐insulin serum pool was 1.71 mU/L.

### 2.3 Sample collection

A total of 19 residual serum samples with insulin concentrations between 200 and 300 mU/L, without bilirubin, hemolysis, and lipemia were collected from Peking Union Medical College Hospital for evaluating the effect of the different matrixes.

### 2.4 Laboratory measurements

Serum insulin concentration was detected using a Siemens ADVIA Centaur XP^®^ automatic chemiluminescence immunoassay analyzer, with its corresponding reagents and calibrators provided by the manufacturer. Calibration was performed according to the manufacturer's instructions. The analytical sensitivity of this assay was 0.5 mU/L. Measurements were performed according to the standard operating procedure (SOP).^12^, ^13^ The instrument was calibrated and prophylactically maintained every year. Our laboratory also participated in external quality assessments by the National Center for Clinical Laboratories and the College of American Pathologists to guarantee the accuracy and reliability of results.

### 2.5 Dilution protocol

The matrixes were divided into four groups for experiments: A (Siemens original diluent); B (pure water); C (0.9% NaCl); D (low‐insulin serum). All of the 19 high‐insulin samples were diluted by four different matrixes. The dilution factors were 1:2, 1:5, and 1:10. Among these, 1:2 and 1:5 dilutions were made using the instrument's automatic dilution procedure, and 1:10 dilution was made by using a two‐fold manual dilution method followed by a five‐fold auto‐dilution using the analyzer.

### 2.6 Statistical analysis

Data were analyzed using Excel 2010 (Microsoft Inc), SPSS 20.0 software (SPSS Inc), and/or Medcalc Statistical software (Broekstraat, Mariakerke, Belgium). Passing‐Babloke regression was used to determine the relationship between the original diluent results and the results obtained with the different dilution matrixes. Bland‐Altman plots were used to compare tests graphically to assess bias. Acceptable bias was determined based on the biological variation of insulin (±15.5%). Pearson correlation coefficients between 0.36 and 0.67 indicate modest or moderate correlations, whereas those between 0.68 and 1.0 represent strong or high correlations.^14^

## 3 RESULTS

### 3.1 Precision

Precision was evaluated according to CLSI EP‐15A. As shown in Table 1, the repeatability ranged from 1.3% to 1.9%, and the within‐laboratory CV (%) ranged from 1.9% to 3.2%.

**TABLE 1:** Precision of serum insulin measurement

QC	Precision	Results
QC_1_	Mean ± SD (mU/L)	16.39 ± 0.52
	Repeatability (%)	1.9
	Within laboratory CV (%)	3.2
QC_2_	Mean ± SD (mU/L)	52.51 ± 1.59
	Repeatability (%)	1.8
	Within laboratory CV (%)	3.0
QC_3_	Mean ± SD (mU/L)	161.14 ± 3.09
	Repeatability (%)	1.3
	Within laboratory CV (%)	1.9

### 3.2 Linear regression between original and dilution‐based results by different matrixes

**FIGURE 1:**
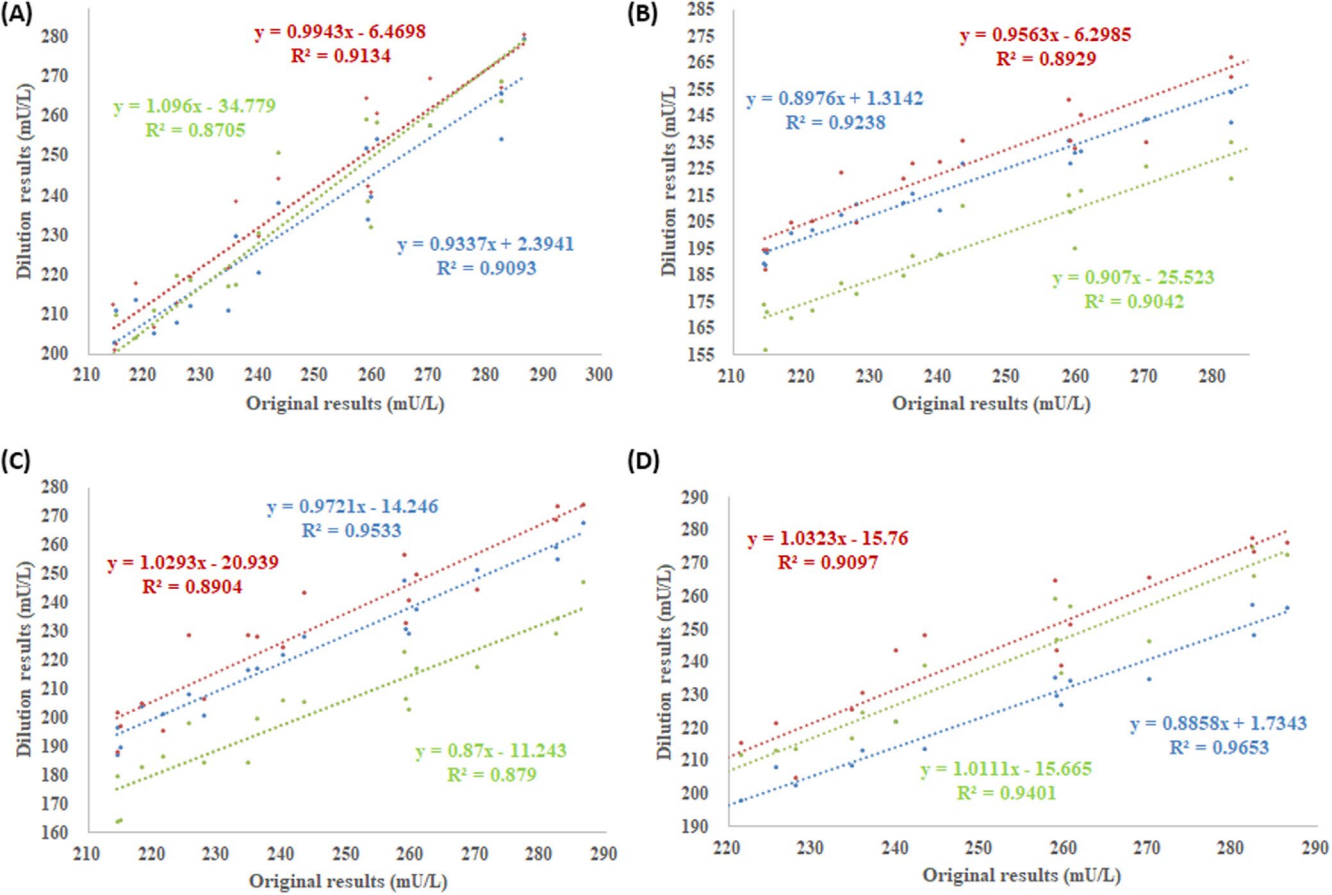
Passing‐Bablok regression of insulin level between the original and dilution results. A to D represent four different dilution matrixes including the original diluent, pure water, 0.9% NaCl, and low‐insulin serum, respectively. The blue line represents 1:2, the red line represents 1:5, and the green line represents 1:10

High‐insulin serum samples were diluted by 1:2, 1:5, and 1:10 using the diluents A to D. The linear regression equation between original (X axis) and dilution (Y axis) results are shown in Figure 1. The linear correlation coefficients of A to D were higher than 0.9. The *R* ^2^ values were 0.871‐0.913, 0.893‐0.924, 0.879‐0.953, and 0.910‐0.965 for A to D, respectively. The 95% CI of slope and intercept contained 1 and 0 in A, B, and C.

### 3.3 Recovery

**FIGURE 2:**
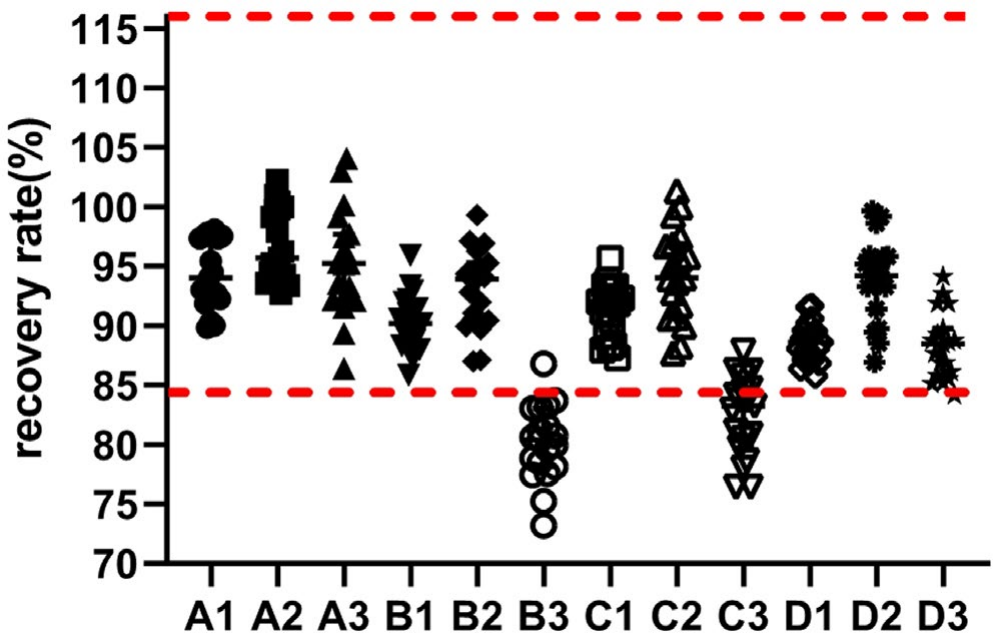
Recovery rates for the original and dilution results. A to D represent four different dilution matrixes including original diluent, water, 0.9% NaCl, and low‐insulin serum. The numbers 1‐3 represent the different dilution factors (2, 5, 10)

Insulin recovery results of the different matrixes are shown in Figure 2 and Table 2. The recovery rates were 86.4%‐104.0% (original diluent), 73.2%‐99.3% (pure water), 76.4%‐101.3% (0.9% NaCl), and 84.2%‐99.7% (low‐insulin serum). Among these, the recovery results of the original diluent and low‐insulin serum were better than those of the others and satisfied clinical requirements.

**TABLE 2:** Recovery results of four different diluents

Original results (mU/L)	Diluent	Dilution results (mU/L)			Recovery (%)		
		2	5	10	2	5	10
214.4‐286.4	A	199.5‐331.6	201.0‐357.0	185.3‐361.5	89.8‐98.1	92.7‐102.3	86.4‐104
	B	188.8‐325.1	186.9‐352.8	157.0‐333.7	85.8‐95.9	87.0‐99.3	73.2‐86.8
	C	187.3‐347.0	188.1‐361.7	163.9‐336.2	87.3‐95.7	87.7‐101.3	76.4‐87.9
	D	185.0‐316.5	180.8‐349.7	196.2‐365.1	85.8‐91.7	86.9‐99.7	84.2‐94.1

Note

A to D represent four different dilution matrixes including the original diluent, water, 0.9% NaCl, and low‐insulin serum.

## 4 DISCUSSION

According to the statistics of the International Diabetes Federation (IDF), the number of patients with diabetes (20‐79 years) in Southeast Asia reached 82 million in 2017, and it is estimated that it will reach 151 million in 2045.^15^ Serum insulin concentration provides a reliable basis for the diagnosis, treatment, and monitoring of many diseases, especially hyperinsulinemia, in which the serum insulin concentration is higher than normal individuals. The determination of serum insulin concentration is one of the most important tests for diabetes typing and evaluating islet function and insulin resistance in diabetes. Moreover, serum insulin concentration is also a qualitative diagnostic method for insulinoma. Insulinoma patients generally have elevated serum insulin on fasting or onset, and when their insulin concentration is normal, OGTT tests show a sharp increase in serum insulin concentration. When the human body is in a state of hyperinsulinemia for a long time, ovarian function also worsens,^16^ further increasing the incidence of ovarian and endometrial cancer.^17^, ^18^ In addition, insulin level also increases in neonatal hypoglycemia. Finally, the clinician also relies on a precise measurement of insulin to determine the peak time in the insulin release test and the rate of insulin increase compared with fasting. Thus, for many reasons, having an accurate measure of serum insulin concentration is necessary.

In this study, we compared the effectiveness of the four matrixes pure water, 0.9% NaCl, low‐insulin serum, and the original manufacturer's diluent, to dilute high‐insulin serum (>300 mU/L). We found that using the original diluent was much better than the other diluents, showing recovery rates of 89.8%‐98.1% (dilution factor 2), 92.7%‐102.3% (dilution factor 5), and 86.4%‐104.0% (dilution factor, 10). The Siemens original diluent's package is 2× 10 mL with a validity period of 21 days, and it can be used for approximately 30 samples (>300 mU/L) for a month according to previous measurement tests. However, this would cause the wastage of 22.72% of the original diluent. Thus, it is not ideal to dilute high‐insulin serum (>300 mU/L) using the original diluent. When using low‐insulin serum to dilute, the recovery rates were 85.8%‐91.7% (dilution factor 1:2), 86.9%‐99.7% (dilution factor, 1:5), and 84.2% to 94.1% (dilution factor, 1:10). However, although these figures are strong, low‐insulin serum is not easy to obtain and also presents biosafety risks.

This study also revealed that the recovery rates of pure water were 85.8% to 95.9% (dilution factor 2), 87.0%‐99.3% (dilution factor 5), and 73.2%‐86.8% (dilution factor 10). For 0.9% NaCl, the recovery rates were 87.3%‐95.7% (dilution factor 2), 87.7%‐101.3% (dilution factor, 5), and 76.4%‐87.9% (dilution factor 10). Based on these results, we conclude that dilution factors 1:2 and 1:5 were much better than 1:10 for both pure water and 0.9% NaCl. Additionally, pure water and 0.9% NaCl are, of course, much easier to obtain.

**FIGURE 3:**
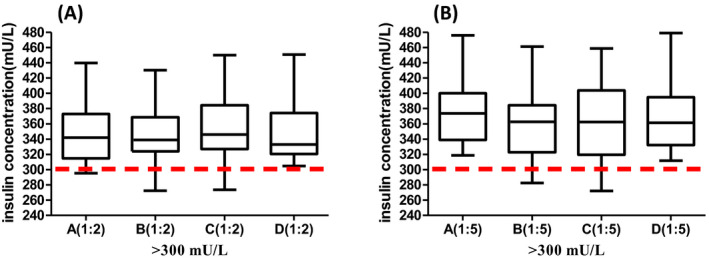
Distribution of samples (>300 mU/L) by different diluents. A to D represent four different dilution matrixes including the original diluent, water, 0.9% NaCl, and low‐insulin serum

To confirm the clinical application of this study, we collected the residual serum samples (>300 mU/L) and used different diluents to determine the actual distribution of samples (>300 mU/L). Sixteen samples with insulin concentrations higher than 300 mU/L were collected from October 2019 to January 2020. Thus, we used the four diluent matrixes to detect the insulin concentration in samples (>300 mU/L). The distribution of insulin concentrations higher than 300 mU/L is shown in Figure 3. Among the 16 samples with insulin concentrations higher than 300 mU/L, only one sample showed a concentration higher than 1500 mU/L after 1:5 dilution. After 1:10 dilution, the insulin concentration of this sample was 1925 mU/L (original diluent), 1535 mU/L (pure water), 1536 mU/L (0.9% NaCl), and 1806 mU/L (low serum samples). Based on the clinical application in combination with the recovery results, we found that the insulin concentration in serum samples (insulin concentration >1500 mU/L) diluted with pure water or 0.9% NaCl to dilute (1:10) was lower than that in samples diluted with the original diluent, while the insulin concentration (>1500 mU/L) diluted using low‐insulin serum was similar to that diluted using the original diluent.

According to clinical experience, most of the high‐insulin serum samples contain less than 1500 mU/L. Making large dilutions of patient samples can introduce error, and laboratories should establish appropriate volumes of sample and diluent to be used to minimize dilution errors. Thus, in this study, it is necessary to dilute high‐insulin serum (>300 mU/L and <1500 mU/L) using 0.9% NaCl, pure water, or low‐insulin serum with dilution factor of 1:5. However, for specific samples (>1500 mU/L), it is better to use low‐insulin serum samples for dilution with a dilution factor of 1:10.

To further increase the applicability of this study, we also compared the linearity, sample diluent, package of sample diluent, and validity of the Siemens products with diluents from Architect, Beckman, and Roche. The linearity values obtained from these diluents were 0.5‐300 mU/L, 1‐300 mU/L, 1‐300 mU/L, and 0.2‐1000 mU/L, respectively. Except for Roche, all these manufactures recommend diluting samples when the serum insulin concentration is higher than the upper limit of linearity. The basic performances of sample diluents from the four manufactures are shown in Table 3. The results indicate that, where insulin reagents are used to measure serum insulin concentrations greater than 300 mU/L for clinical application, evaluation of the effects of different diluents should be considered to optimize cost and effectiveness.

**TABLE 3:** Basic performance of sample diluents from various manufacturers

	Linearity (mU/L)	Higher than upper limit	Original diluent	Package	Validity (d)
Siemens	0.5‐300	Dilution	Insulin diluent	2× 10 mL	21 d
Architect	1‐300	Dilution	Multi‐assay, manual Diluent	100 mL	16 M
Beckman	1‐300	Dilution	Access Sample Diluent A	32.9 mL	56 d
Roche	0.2‐1000	NA	NA	NA	NA

## 5 CONCLUSION

From the perspective of health economics, this study confirms that high‐insulin serum (higher than upper limit of linearity) can be diluted by pure water, 0.9% NaCl, or low‐insulin serum on the Siemens ADVIA Centaur XP^®^ instrument, but the dilution factor should be 1:5 or lower. In order to standardize the operation, we recommend using 0.9% NaCl to dilute high‐insulin serum (>300 mU/L) with a dilution factor of 1:5 on the Siemens ADVIA Centaur XP^®^ instrument. Under these conditions, the clinically reported range is 0.5‐1500 mU/L, which meets clinical requirements. However, for specific samples (>1500 mU/L), it is better to use low‐insulin serum samples for dilution with a dilution factor of 1:10.

## CONFLICT OF INTEREST

There was no conflict of interest.

## ETHICAL APPROVAL

This study was approved by the Ethics Committee of Peking Union Medical College Hospital of the Chinese Academy of Medical Sciences. As this study was retrospective in nature and the results were anonymized, informed consent for the use of samples was not required.
